# Arterial Stiffness and Adult Onset Vasculitis: A Systematic Review

**DOI:** 10.3389/fmed.2022.824630

**Published:** 2022-05-12

**Authors:** Alberto Lo Gullo, Clemente Giuffrida, Carmela Morace, Giovanni Squadrito, Paola Magnano San Lio, Luisa Ricciardi, Carlo Salvarani, Giuseppe Mandraffino

**Affiliations:** ^1^Rheumatology Unit, Department of Medicine, ARNAS Garibaldi, Catania, Italy; ^2^Emergency Unit, Department of Emergency Urgency Unit, IRCCS Neurolesi Bonino Pulejo - Piemonte, Messina, Italy; ^3^Internal Medicine Unit, Department of Clinical and Experimental Medicine, University of Messina, Messina, Italy; ^4^Department of Clinical and Experimental Medicine, University of Catania, Catania, Italy; ^5^Allergy and Clinical Immunology Unit, Department of Clinical and Experimental Medicine, University of Messina, Messina, Italy; ^6^Rheumatology Unit, Department of Internal Medicine, University of Modena and Reggio Emilia, Modena, Italy AUSL-IRCCS, Reggio Emilia, Italy

**Keywords:** vasculitis, inflammation, atherosclerosis, arterial stiffness, Behcet disease, Takayasu arteritis, ANCA vasculitis

## Abstract

Chronic inflammation represents the cornerstone of the raised cardiovascular (CV) risk in patients with inflammatory rheumatic diseases (IRD), including vasculitis. Standardized mortality ratios in these patients are higher as compared to the general population, and the excess of premature mortality is due to early atherosclerotic events. Thus, IRD patients need appropriate CV risk assessment and management according to this CV disease (CVD) burden. Adequate control of CV risk is still lacking in usual care, but early diagnosis of silent and subclinical CVD is crucial to improve the long-term prognosis of these patients. Increased arterial stiffness may provide a pathophysiological link between inflammation and increased cardiovascular risk. Several noninvasive methods are now available to estimate artery stiffness in the clinical setting, including pulse wave velocity assessment. The independent predictive value of arterial stiffness for cardiovascular events has been demonstrated in general as well as in selected populations, and reference values adjusted for age and blood pressure have been suggested. Thus, arterial stiffness is an interesting biomarker for cardiovascular risk stratification. This systematic review summarizes the additional value that PWV measurement can provide in the setting of vasculitis, with a focus in the different clinical stages and CV risk prevention. This systematic review is registered with registration number: Prospero CRD42021259603.

## Introduction

Patients with chronic inflammatory rheumatic diseases (IRD) including systemic lupus erythematosus (SLE), rheumatoid arthritis (RA), connective tissue disease, and also vasculitis have increased risk of developing premature CVD (1–3). The pathogenesis of CVD in these conditions is multi-factorial, and is thought to result from an interaction among inflammation, metabolic factors, therapies and disease-related factors (4). Emerging evidence suggest that the two classical pathways of arterial damage, namely, atheromatosis (i.e., atheromatic plaque formation), and arteriosclerosis (i.e., arterial stiffening), are accelerated, thus participating in the development of microvascular and macrovascular complications in rheumatic disease (5).

Remarkably, atherosclerosis in patients with IRD is often characterized by higher plaque vulnerability; Karpouzas and coll. showed that patients with RA present with a greater coronary atherosclerotic burden, and higher prevalence of more vulnerable non-calcified and mixed plaque compared with controls (6). Moreover, vulnerability and the risk of plaques rupture are characteristics ascribed to disease activity, as well as the risk of thrombosis in IRD (7).

The primary systemic vasculitis are rare autoimmune diseases potentially resulting in life-threatening organ ischemia and infarction; they are characterized by idiopathic inflammation of blood vessel walls and are classified by size of blood vessel affected (8). Although death typically occurs prematurely as a consequence of either uncontrolled vasculitis or infection due to immunosuppression, atherosclerotic cardiovascular disease (ASCVD) is now the leading cause of mortality in these patients (9, 10).

### Atherosclerosis and Vasculitis

Potential mechanisms underlying the accelerated atherosclerosis in systemic vasculitis also include the infiltration of activated inflammatory cells within the affected artery wall (11). In addition, different mediators including metalloproteinase, VEGF and PDGF are increased in vasculitis, thus contributing to intimal hyperplasia and luminal narrowing (11). Increased plasma levels of CRP as well as other pro-inflammatory cytokines lead to pro-atherogenic profile, through the increased expression of adhesion molecules, cell recruitment, and smooth muscle cell stimulation and macrophage apoptosis, enhance atherogenesis (12). Inflammatory cytokines also affects coagulation via thrombomodulin-C (11). In addition, autoantibodies such as anti-endothelial cell, anti-cardiolipin, and MPO-ANCA, may contribute in damaging endothelial cell, leading to a pro-thrombotic state. These antibodies may directly activate TNF-primed neutrophils, leading to generation of reactive oxygen species and subsequent endothelial damage; immune complexes further contribute to pathogenesis by fixing complement and by binding to neutrophil Fcg receptors and activating neutrophils (13). The formation of neutrophil extracellular traps (or NETosis), which play a key role in increasing inflammation and autoantibody production in ANCA-associated vasculitis, also seems to have a crucial role in initiating macrophage activation in atherosclerosis (14). Furthermore, the vasculitic injury of arterial wall may accelerate atherosclerosis and may alter arterial anatomy perturbing arterial blood flow and leading to a pro-inflammatory status on vascular endothelium. Even therapies could affect the endothelium homeostasis. Despite certain targeted immunosuppressive drugs seems to reduce the risk of CVD, glucocorticoids could have detrimental metabolic effects, promoting weight gain, hypertension, dyslipidemia and hyperglycemia (15), further exacerbating vascular dysfunction and promoting atherosclerotic process. Lastly, traditional CV risk factors are more prevalent in vasculitis, with increased rate of target organ involvement. Microbiome is currently investigated in order to assess its role in both atherosclerosis (16) and the systemic vasculitis (17); potential mechanism involved are the stimulation of the immune system, and the increased availability of certain pro-atherogenic metabolites including trimethylamine-N-oxide. RA in remission shows lower values of laboratory inflammatory markers, lower blood pressure, and better arterial compliance; moreover, MTX has been associated with lowered rates of CVD in different studies, and TNF inhibitors may protect against CV events in patients with RA (18). Furthermore, the recent successes of targeted anti-inflammatory medications including canakinumab and colchicine in reducing the incidence of cardiovascular events in patients with atherosclerosis provide strong support for the critical role of inflammation in atherosclerosis pathogenesis (19, 20).

### Arterial Stiffness

Patients at high risk of CV events could be stratified with the use of noninvasive surrogate markers of CVD (21), such as carotid US, particularly those included in the category of moderate CV risk according to risk chart algorithms. In addition to maintaining a tight control of the rheumatic disease, looking for clinical remission and management of traditional CVRFs such as dyslipidemia and hypertension should be routinely assessed in patients with IRD.

Increased arterial stiffness (AS) is one of the earliest stages of the atherosclerotic process (22, 23), and PWV is widely accepted as an accurate and non-invasive method to assess AS in humans (24). While PWV is a direct measure of arterial distensibility, the AIx is a more complex parameter depending on vascular elasticity and peripheral resistance (25). Arterial wall rigidity is considered an independent predictor of all-cause and cardiovascular mortality in several clinical settings, including hypertensives, end-stage renal disease, dyslipidemic, in elderly people, and also in IRD patients (26–28).

Mechanic properties could be altered long before the appearance of clinical lesions, reflecting alterations in the arteries structure and affecting their functional features. The pulse wave velocity (PWV) is measures the travel speed of the pulse pressure along a segment of the arterial tree; the augmentation index (AIx) is defined as the increment in pressure after the first systolic shoulder to the peak of the aortic pressure expressed as a percentage of aortic pulse pressure (29).

To assess AS indices some features are generally required. The distance between the recording sites and the suprasternal notch are measured using a tape measure. Electrocardiography is used to determine the start of the pulse wave. The PWV is determined as the differential time between the 2 different recording sites divided by the travel distance of the pulse waveform. Several methods and technologies have been proposed to evaluate AS parameters, including PWV and AIx. The classical “two-points” evaluation was long considered the—noninvasive—gold standard to assess PWV (30); later, semi-automated tools were developed to assess more easily PWV and/or AIx. Of these, the SphygmoCor^TM^ CVMS (AtCor Medical, Sydney, Australia) uses a tonometer and 2 different pressure waves obtained at the common carotid artery (proximal recording site) and at the femoral artery (distal recording site); the Arteriograph^TM^ (TensioMed, Budapest, Hungary) -applied to the upper arm- explores the time difference between the first and second waves originates from the aortic bifurcation and the sum of travel time of the pulse in the descending aorta forward and backward; the Complior^TM^ (Artech Medical, Pantin, France) system uses two sensors simultaneously exploring carotid and femoral waveforms, thus estimating cfPWV by dividing the distance separating the two sensors by the time corresponding to the period separating the start of the rising phase of the carotid pulse wave and that of the femoral pulse wave. Also, AS tracking systems could implemented in the ultrasound machine(s) (30–33).

PWV typically increases from the proximal aorta (3–4 m/s), through the descending aorta (5–6 m/s), the ilial-femoral segment (7–8 m/s), to the foot (9–10 m/s) or hand. The wall-to-lumen ratio and the amount of collagen relative to elastin are major contributors to this heterogeneity of PWV in the human circulation. Increasing the wall-to-lumen and/or the collagen to elastin ratios *favor* increased stiffness (34–37). Also, AS independently predicts death (from all causes and from cardiovascular causes in particular) and CV outcomes in healthy elderly people, diabetic patients, hypertensive patients, general adult populations such as those sampled by the Framingham Study, and patients with end-stage renal disease (35, 38).

Several factors, including cardiac performance, structural elements in the vessel wall and rheological characteristic of the blood, are recognized as determinants of the arterial stiffness. Arterial stiffening reflects the degenerative changes of ECM in the media layer, and is characterized by elastin fatigue fracture and collagen deposition and cross-linking. Arterial stiffening differ from atherosclerosis, a process that typically involves the intima layer and is characterized by lipid accumulation, inflammatory cells and vascular smooth muscle cell migration, and foam cell development. On the other hand, both processes often coexist in the same vascular *territories*, share some mutual risk factors, and are part of the vascular aging process. Furthermore, experimental studies have shown that changes in ECM proteins and in mechanical properties of the vessel wall may activate some pathophysiologic mechanisms involved in the atherosclerotic process, and clinical studies have demonstrated an independent association between AS and atherosclerotic load, as well as between AS and risk of incident cardiovascular events (39). AS is determined principally by age and blood pressure (30, 40) that may account up to 70% of its variance (36). Chronic inflammation is another pathological condition involved in both arterial stiffening and atherosclerosis (34). Altogether, these evidences suggest AS as a promising biomarker for cardiovascular risk stratification.

With this systematic review we aimed to describe the potential usefulness to estimate AS indices in vasculitis in relation to disease activity, also to better identify patients at high cardiovascular risk.

## Methods

This research was performed following methods that are reported in the PRISMA Statement, and the systematic review is registered with registration number: Prospero CRD42021259603. Two authors (ALG, GM) independently searched published studies that were indexed in MEDLINE and EMBASE from January 1990 to May 2021. If needed, a manual search was performed according to the citation lists of the relevant literature. The following key words were used: (Takayasu arteritis[Title/Abstract] OR Takayasu's arteritis [Title/Abstract] OR pulseless disease[Title/Abstract] OR aortitis syndrome[Title/Abstract] OR Behçet syndrome[Title/Abstract] OR Behçet disease[Title/Abstract] OR Behçet's disease[Title/Abstract] OR antineutrophil cytoplasmic antibody-associated systemic vasculitis[Title/Abstract] OR ANCA associated vasculitis[Title/Abstract] OR granulomatosis with polyangiitis[Title/Abstract] OR eosinophilic granulomatosis with polyangiitis[Title/Abstract] OR microscopic polyangiitis[Title/Abstract] OR Churg-Strauss syndrome[Title/Abstract] OR Wegener's granulomatosis[Title/Abstract]) which were combined with (arterial stiffness[Title/Abstract] OR artery stiffness[Title/Abstract] OR vascular stiffness[Title/Abstract] OR pulse wave velocity[Title/Abstract] OR pulse wave analysis[Title/Abstract] OR pulse wave[Title/Abstract] OR carotid stiffness[Title/Abstract] OR aortic stiffness[Title/Abstract] OR pulse pressure[Title/Abstract]).

The studies were restricted to those written in the English language. The list of titles and abstracts was initially screened for relevance by the two review authors (ALG and GM). We then selected original articles that were full-length publications in peer-reviewed journals. In addition, the reference lists of selected articles were also manually reviewed. Any disagreements were resolved by discussion, or by involving a third review author (CM). Articles were excluded if any of the following criteria were present: (a) lacking of a healthy control group; (b) article based on animal studies; (c) article based on *in vitro* or experimental model-based studies; or (d) review (e) case report (f) poster abstract.

The preliminary search by using the research strategy retrieved 58 articles. Out of these, 27 articles were excluded after judgment of their eligibility according to screening of titles and abstracts; in details, 7 articles were excluded as they were case-reports, 6 articles due to lacking of investigation on AS, 4 due to no full text available (“poster abstract”, “focus imaging”, “letter”, “comment on”), 8 as they were review articles, 1 study was a study protocol for a randomized controlled trial, 1 study included pediatric patients. A total of 31 studies were eligible for the present systematic review.

## Results

### Takayasu Arteritis

Takayasu's arteritis (TAK) is a chronic vasculitis of unknown etiology with a predilection for the major elastic arteries such as the aorta, its main branches and the pulmonary arteries (41, 42). The media and the adventitia of the arterial walls are predominantly involved, with intimal thickening and arterial occlusions as important late phenomena (42, 43). During the early active stage of the disease, arterial biopsy shows granulomatous inflammation and patchy destruction of the medial musculoelastic lamellae (41, 44). Later the microscopic changes become nonspecific and consist of sclerosing arteritis, fibrous intimal hyperplasia, medial scarring and adventitial fibrosis (43, 45). TAK patients had a higher prevalence of cardiovascular risk factors, and dramatically experienced more CVE, when compared to the general population (46, 47). Inflammatory response and platelet hyperactivity both contribute to increasing the risk of acute CVE (48, 49). Coronary artery disease has been reported to be present in 10–20% of TAK patients, and cerebrovascular disease could occur in 10% of TAK patients (50, 51).

For the first time, Raninen reported in 2002 that 16 patients with TAK presented with an increased AS with respect to 16 age and sex matched controls. They used Peterson's elastic modulus (that estimates vascular stiffness without taking into account the influence of wall thickness), the Young's modulus (that may reflect the true elastic characteristics of the arterial wall better because wall thickness is included in the calculation) and thee stiffness index beta as markers of AS, in order to obtain a pressure-independent estimation of arterial distensibility. All indices of carotid artery stiffness were increased in the TAK group as compared to healthy subjects. The indices of femoral artery stiffness were also higher in patients with TA, even though the difference in the stiffness constant b was not statistically significant (52).

Ng enrolled 10 patients with TAK and 11 woman as healthy controls. Mean PWV-CF was higher among TAK patients. In contrast, there was no difference between the two groups in PWV-CR. The mean estimated carotid AI and aortic AI derived from the radial artery was higher in TAK patients compared with controls. Both PWV values did not correlate with CRP or ESR, nor with clinical disease activity. No difference between active and inactive disease regarding PWV was reported; indeed, this could be due also to the diagnosis of TAK, often late, when an irreversible structural damage to the vasculature is already established. Furthermore, the available clinical criteria of disease activity, including CRP and ESR, may be not so accurate. The multiple regression model estimating the dependence of PWV-CF from SBP, DBP, BMI, and also the diagnosis of TAK, suggested DBP, BMI and TAK as potential contributors, but not SBP. Accordingly, TAK seems to contribute significantly to the increase in PWV-CF (53).

Salles Rosa Neto analyzed CF-PWV in 27 female patients compared to 27 controls, reporting increased AS in TAK disease. PWV values were not correlated with ESR, CRP, cumulative dose of steroid, ejection fraction, or lipid levels; indeed, vascular procedure only was significantly associated with CF-PWV, whereas no association was observed as regards disease activity, history of HTN, or disease duration. The multivariate linear regression model showed that age, mean BP, and TAK explained the 93.8% variability of the PWV (54).

Liu et al. recruited seventy-two patients with TAK. Twenty-four patients were classified into the high-ba-PWV group. BMI, SBP, DBP, mean blood pressure, plasma NT-proBNP levels and total cholesterol levels were significantly higher in the high-baPWV group than in the low-ba-PWV group. Ba-PWV values were significantly higher in the patients with active disease than in those in remission. However, there were no significant correlations between the ba-PWV values and inflammatory markers. A stepwise multiple linear regression analysis showed that the mean blood pressure, age, and BNP levels were independently associated with the ba-PWV values in TAK after adjusting these parameters for the body mass index, total cholesterol level and use of calcium channel blockers and statins (55).

Wang et al. evaluated 48 TAK coronary artery disease patients, and they found increased ba-PWV in TA-related CAD as compared with CAD patients. CAD patients had atherogenic lipid profiles, including higher levels of low-density lipoprotein cholesterol. In the multiple regression analysis ba-PWV was independently associated with the severity of TAK in patients with coronary artery involvement, even after adjusting the confounding factors (such as age, BMI, total cholesterol, and systolic blood pressure). Multiple linear regression analysis suggested ba-PWV as independent predictor of the extent of CAD, assessed by SYNTAX score in TAK patients. In the multivariate logistic regression analysis, the significant independent determinant of in-stent restenosis was a ba-PWV of 17.00 m/s or higher; the multivariate Cox proportional hazards model confirmed a ba-PWV of 17.00 m/s or higher an independent predictor of MACE. Authors concluded that increased AS assessed by ba-PWV would be of great clinical value to identify TAK patients with drug-eluting stent who have a high risk for in-stent restenosis and MACE (56).

Yang et enrolled 15 tan patients and 15 matched controls; the patients with TAK had a higher PWV-CF value measured by echocardiography, compared with healthy controls. The echocardiographic measured PWV-CF was significantly dependent on the TAK, age and pulse pressure. PWV-CF did not correlate with the echocardiographic measured cardiac systolic and diastolic parameters and the laboratory variables in TAK patients (57).

Yurdakul included 33 patients with TAK, 18 patients with SLE; and 20 age- and sex-matched control subjects. Aortic strain and distensibility were decreased, whereas aortic stiffness was markedly increased in patients with TAK. There was no difference in aortic strain and stiffness measurements between the SLE group and the control group, while aortic distensibility was impaired in both groups (58).

He et al. analyzed 240 patients with TAK of which 74 had cardiovascular disease. They found that increased ba-PWV was independently associated with CVE and the strongest determinants for ba-PWV in TAK were age, angiographic type V, mean blood pressure, renal dysfunction, hyperlipidemia. The ROC curve analysis estimated 16.26 m/s as optimal cut-off value of ba-PWV for CVE (area under the curve: 0.672, 95% CI: 0.594–0.750, *p* < 0.001; sensitivity and specificity were 45.9 and 83.7%, respectively). Increased ba-PWV was independently associated with CVEs in patients with TAK, therefore according to this study higher ba-PWV may be a potential marker to predict CVE in TAK (59).

In another study 67 patients with TAK and 67 age and sex matched healthy controls were recruited. Patients with TAK were grouped according to disease activity. ba-PWV was significantly higher in the patients with TAK than in the healthy subjects, and it was also significantly higher in the patients with inactive TAK than in the healthy subjects; moreover, ba-PWV was significantly higher in the patients with active TAK than the patients with inactive TAK. In the multiple linear regression analysis estimated with ba-PWV as dependent variable, TAK, and MAP were significantly associated with ba-PWV also after adjusting for confounder (age, SBP, DBP, PP, BMI, HR, total cholesterol, HDL, and LDL). No significant associations between ba-PWV and ESR or CRP were found in overall patients with TAK, and in patients with active or inactive TAK. However, in patients with TAK without immunosuppressive therapy, ba-PWV was significantly correlated with CRP, but not with ESR. The AS as measured by ba-PWV is significantly increased in patients with TAK, likely correlated with systematic inflammation, and it is significantly associated with TAK disease activity probably serving as an independent predictor of active TAK (60).

In conclusion, all the above studies reported an increased arterial stiffness in TAK and in some studies one of the principle factors influencing the arterial stiffness measurement was the disease itself proving a role of inflammation in accelerated atherosclerotic process in this conditions.

### ANCA Vasculitis

ANCA-associated vasculitides (AAV) are a heterogeneous group of systemic diseases characterized by inflammation of small- and medium-sized vessels, variably associated with ANCA directed against PR3 or MPO (61, 62). Among AAV, GPA (formerly Wegener granulomatosis) and MPA are the two most common subtypes and, together with EGPA, (formerly Churg-Strauss syndrome) account for an estimated combined prevalence of 42.1 per 100,000 adult population in the United States (63). A well-established long-term complication of many inflammatory diseases is premature atherosclerosis and CVE (64). A high incidence of cardiovascular events has also been reported in AAV (65, 66); therefore, the most recent EULAR guidelines for AAV recommend periodic assessment of cardiovascular risk in AAV patients (67).

Booth (68) enrolled 31 patients (15 with active AAV) and the 32 matched controls; disease subgroups included Wegener's granulomatosis (n 23), microscopic polyangiitis (n 4), and Churg-Strauss disease (n 4). AIx and PWV were higher as compared to controls, and both these AS parameters were correlated to CRP. PWV was positively associated with increasing age and blood pressure, whereas AIx was positively associated with female sex and MAP, but negatively associated with heart rate. No correlation was found between AS parameters and ANCA levels, disease duration, organ involvement and severity (serum creatinine), or prednisolone dose.

Yildiz (69) enrolled 5 patients with GPA and reported that PWV-CF were increased in patients with GPA as compared with control group. Although they found a positive correlation between PWV and heart rate, they did not find any significant correlation between PWV and anthropometric or other hemodynamic parameters. In addition, they described a positive correlation between PWV and ESR in patients with GPA.

In another study from Netherland, 40 ANCA vasculitis patients were enrolled and compared to 38 controls. Femoral PWV was comparable between AAV patients and controls, as was radial PWV. However, when PWV values were corrected for MAP, femoral PWV was higher in AAV patients. In addition, radial MAP-corrected PWV was higher in patients with AAV. Furthermore, PWV measurements did not differ between patients with a high or low percentage of CD4+CD28^null^ T cells (70).

CD4+ T cells not co-expressing the co-stimulatory molecule CD28 (CD4+CD28^null^) are acknowledged as potential players in accelerating the atherosclerotic processes, also in patients with AAV; this subset of T cells in fact was found preferentially in unstable rather than stable atherosclerotic plaques, and also they have been shown to exhibit endothelial cytotoxicity in the context of acute coronary syndrome and AAV in *in vitro* assays. These evidences may suggest a direct involvement in plaque disruption (71).

Chanouzas et al. (72) enrolled 56 patients diagnosed with ANCA vasculitis, of which 34 were PR3 positive, and 18 MPO positive. Also for this study, CD4^+^CD28^null^ T cells were evaluated. The univariable analysis showed that age, percentage of CD4^+^CD28^null^ T cells, plasma concentration of TNF, and blood pressure parameters were associated with increased PWV. Furthermore, the multivariable linear regression model demonstrated that the percentage of CD4^+^CD28^null^ T cells were associated with increased AS independently of age, proteinuria, peripheral MAP, and plasma concentration of TNF. A PWV increase of 0.66 m/s for each 10% increase in CD4^+^CD28^null^ T cells was reported. This relationship did not change when systolic blood pressure or pulse pressure was replaced by MAP, and the size of the CD4^+^CD28^null^ T-cell expansion remained independently associated with increased PWV.

Forty four patients (21 men and 23 women) diagnosed with GPA and 53 controls matched for age, sex, BMI and typical risk factors for cardiovascular diseases (22 men and 31 women) were enrolled in the study by Pacholczak et al. (73). Aortic stiffness was similar between GPA patients and controls, and it was negatively associated with blood leukocyte count and CRP levels. Comorbidities and medication had no impact on aortic stiffness.

In conclusion, a definite role of AS measurements in ANCA vasculitis is not clear, in fact only in two studies AS values were higher compared to controls, in particular after adjusting for confounders while in others studies AS was similar to controls. None of the above-mentioned trials analyzed the impact of the disease activity nor the early and late phase of the disease in assessing AS or the limited and diffuse GPA, therefore further studies are needed to assess AS in ANCA vasculitis.

### Behcet Disease

Behcet's syndrome (BS) is a chronic, multisystem disorder characterized by genital and oral aphthae, skin lesions, and uveitis (74). BS is characterized by the contemporaneous involvement of both arteries and veins of all sizes, and presents a unique tendency for aneurysm formation (75). Within BS, patients suffering from recurrent inflammatory thromboses involving the venous and, more rarely, the arterial vasculature constitute a specific cluster, called the “vascular cluster” or “Angio-Behcet” (76). Arterial involvement is considered an uncommon vascular feature of BS, although BS could induce aneurysms affecting peripheral, visceral and pulmonary arteries. The simultaneous occurrence of arterial pulmonary aneurysms and peripheral venous thrombosis is the hallmark of Hughes–Stovin syndrome, which is to date considered by some authors as a clinical variant of Angio-Behcet (75, 77). The vascular involvement in BS has a major impact on morbidity and long-term mortality, and has been identified as the leading cause of death in these patients (78).

Kurum et al. (79) analyzed 14 patients with Behcet matched with 28 controls; oral aphthae (in 14 patients, 100%), genital ulcers (11, 84.6%), erythema nodosum (7, 50%), uveitis (7, 50%), arthritis (5, 35%), deep venous thrombosis (4, 33.3%), and neurologic involvement (3, 23.3%) were detected over the entire disease duration. Similar values of PWV were found in patients and controls; in addition, no correlation between duration of disease and PWV was found. Differences of mean PWV of the patients who did and did not have genital ulcers or erythema nodosum or eye involvement or deep vein thrombosis or neurologic involvement were not found to be statistically significant.

Protogerou et al. (80) selected 47 patients made up the study population, 11 of whom had active BD, defined as having at least two symptoms according to the ISG criteria. No sign of clinically active vascular disease was present in any patient at the time of the vascular tests. Subjects with active BD (*n* = 11) had lower AIx and central systolic blood pressure (CSBP), but similar peripheral blood pressure, stroke volume, and slightly higher local aortic stiffness in comparison to patients with inactive BD (*n* = 36). Lower AIx was found in patients with active BD compared to those with inactive disease; we also found that AIx in patients with inactive BD had a trend to be higher compared to the control group. The differences in central SBP and AIx were not affected after adjustment for age, sex height and heart rate.

Tunc et al. (81) included 26 patients with BD compared to 20 controls, finding beta aortic stiffness values higher than controls.

Rhee et al. (82) enrolled 41 patients with Behcet matched with 53 controls. All patients with Behcet had an increased beta stiffness compared to controls; furthermore, patients with peripheral arthritis exhibited a higher Beta stiffness than those without peripheral arthritis. In addition, a positive relationship between age of onset and beta stiffness was also noted in linear regression analysis.

Protogerou et al. (83) reported that aortic stiffness evaluated by AI in patients with Behcet was similar to the control group; however, BD patients taking corticosteroids showed values lower than those without corticosteroids and similar to controls, while BD patients not taking corticosteroids showed aortic AI values higher than controls. The negative association between corticosteroids administration and aortic AI was maintained also after adjustment for heart rate, age, gender, blood pressure, reflected wave time transit, height, and cholesterol. Authors suggested a role of inflammation or immuno-modulatory mechanisms in the regulation of pressure wave reflections.

Kobacay et al. (84) found that PWV was higher in rheumatoid arthritis, systemic lupus erythematosus, and Behcet's disease groups as compared to the control group. However, when all variables were included in the regression analysis only age was found to affect PWV independently.

Caldas et al. (85) found that 23 BD patients had significantly higher PWV values as compared with 23 controls. Moreover, the 15 BD patients presenting with systemic disease had PWV values significantly higher than those with exclusive muco-cutaneous manifestations. BD patients with vascular involvement had higher total and LDL cholesterol levels, but similar PWV compared to those without vascular involvement. The bivariate analysis within the BD group demonstrated significant correlations between PWV and systolic and diastolic blood pressure, BMI, total cholesterol, and triglycerides, but triglycerides only were independently associated to PWV in the multivariate linear regression analysis.

Balta et al. (86) evaluated PWV in 36 patients with Behcet compared to 35 controls. AS was higher in patients with BD compared with control group and AS correlated positively with age, the duration of disease, BMI, total cholesterol and Mean Platelet Volume levels in patients with BD.

Yilmaz et al. (87) recruited 96 patients with BD. Each subject was evaluated in active and inactive disease periods. For the control group, 54 healthy age- and sex-matched subjects were enrolled. 24-h PWV was positively correlated with age, duration of BD, weight, BMI, fasting blood glucose, total cholesterol, and LDL-C values. Linear regression analysis assessed that 24-h PWV was positively correlated with age and duration of BD. There were statistically significant differences between the control group and patients with inactive and active BD in terms of 24-h PWV, day PWV, night PWV, day central DBP in this study (*p* < 0.05). No significant difference between the control group and patients with inactive and active BD in terms of 24-h MBP, central SBP, central DBP, and AIx in this study (*p* > 0.05). Patients with active BD had higher PWV values than patients with inactive BD and the controls. According to the vascular function parameters of patients with active and inactive BD, 24-h PWV (6.16 ± 1.26 vs. 5.58 ± 0.73, *p* < 0.012), day PWV (6.22 ± 1.27 vs. 5.63 ± 0.74, *p* < 0.011), and night PWV (6.06 ± 1.30 vs. 5.49 ± 0.74, *p* < 0.015) were higher in patients with active BD than in patients with inactive BD. Other vascular function parameters did not differ between the two groups. This may be explained by more prominent inflammatory changes in the vascular wall in the active disease period.

Celik et al. (88) enrolled 96 BD patients and 60 controls. They evaluated the 24 h profile of blood pressure, AIx and PWV, finding that worse PWV an AIx indices were correlated with the non-dipping status. Authors concluded that non-dipping status and AS may concur to exacerbate the harmful effects on cardiovascular system also in BD, and that these aspects should not be overlooked during the follow-up evaluations of patients with Behcet's disease in addition to conventional risk factors.

Yildirim et al. (89) enrolled 30 patients with BD compared to 30 controls. PWV was 6.35 ± 1.05 m/s in BD group and 5.75 ± 0.83 m/s in control group, and the difference between the two groups was statistically significant. In addition, they found no difference regarding PWV in patients with systemic disease compared to patients with muco-cutaneous involvement. Even in the absence of major atherosclerotic risk factors, BD patients might be at a higher risk for development of atherosclerosis and endothelial dysfunction compared with healthy subjects.

Yolbas (90) compared 49 BD patients to 64 rheumatoid arthritis and 40 controls. The author did not find any difference regarding beta stiffness, also considering active and non-active BD patients or BD patients actively smoking, and there was no correlation with disease activity. In addition, patients with a pathergy reaction have lower arterial distensibility, Young and Peterson elastic modulus.

Ozdemir (91) enrolled 68 patients compared to 40 controls. The authors further divided BD patients according to homocysteine levels in group 1: with homocysteine >15 μmol/L and group 2: homocysteine <15 μmol/L. Both BD patient subgroups beta stiffness indices higher than controls (group 1: 3.73 ± 0.45 and group 2: 3.33 ± 0.24 vs. healthy control group: 3.07 ± 0.17, *p* < 0.001, respectively). Homocysteine level was positively correlated with carotid beta stiffness index (*r* = 0.769, *p* < 0.001), c-IMT (*r* = 0.565, *p* < 0.001) and disease duration.

Ozisler and Kaplanoglu (92) included a total of 33 BD patients (19 women and 14 men); the control group consisted of 33 sex-matched healthy individuals aged 23–58 years. The AS indices (left, right, and mean α- and β-stiffness indices, and the right, left and mean PWV values) they reported were significantly higher in the patient group; moreover, they found higher values in patients with major organ involvement with respect to those with muco-cutaneous involvement, although this difference was not statistically significant. They reported no significant correlation between ESR or CRP and AS parameters, both in the patient and control groups, and also when patients were subdivided according to systemic or muco-cutaneous involvement. Patients with muco-cutaneous involvement were assuming colchicine only, while patients with major organ involvement were receiving immunosuppressive therapy. Therefore, inflammatory status could be different according to treatment needs.

Ayar et al. (93) included 54 patients with BD (27 with Short Disease Duration—SDD and 27 with Long Disease Duration—LDD), and 34 healthy age and sex matched subjects. BSAS scores were not statistically different in patients with BD with SDD or LDD. AIx was significantly higher in all patients with SDD or LDD BD, as compared with controls. However, PWV values were reported not different between BD and controls. When patients with BD with SDD and LDD were compared with each other, PWV was significantly higher in patients with BD with LDD. There was a moderate correlation between PWV and disease duration. AIx was higher in patients with BD than controls regardless of disease duration.

Zencirkiran Agus et al. (94) enrolled 50 BD patients and 49 controls. Carotid-femoral (aortic) PWV was increased significantly in patients with BD as compared with control group, there were no connection between PWV and clinical manifestations. PWV was correlated with age, diastolic BP, mean BP, waist, waist/hip ratio and heart rate in patients with BD, but not with disease duration.

PWV, an ideal indicator of arterial stiffness, is increased in patients with Behcet's disease compared with the controls; furthermore, seem that some features of the disease for example active arthritis could influence AS status. In these studies authors found difference values of AS in patients with active or nor active disease or with long disease duration. Moreover, patients treated with steroids have a lower AI compared to non-treated patients. This data confirm the role on inflammation in driving atherosclerosis in BD. Prospective trials in a large population should be carried out to evaluate the prognostic implications of increased arterial stiffness in BD.

## Strenghts and Limitations

The strength of this review is the evaluation of several studies regarding arterial stiffness in vasculitis covering a long period of time. Moreover, we have pointed out that the measurement of arterial stiffness is a valid method to determine the atherosclerotic burden in patients with vasculitis and it could have a potential role as a screening test also in patients with rheumatic conditions. In fact, PWV might be a easier-to-assess and reproducible marker of early atherosclerosis.

The limitations is that PWV and AIx measurement may be influenced by many confounding factors, significantly limiting reproducibility of arterial stiffness assessment and, in turn, the relevance of our results; in fact the heart rate may affect results, and, with the only exception of AIx@75, all the other outcomes have not been standardized to a specific heart rate. In general population PWV is a surrogate of clinical CV events but in patients with vasculitis, there are no data to suggest whether PWV is a good surrogate of future CVD events. Moreover, the differences among assessment techniques and devices could limit the validity of arterial stiffness parameters as markers of early atherosclerosis and therefore caution is necessary in overall results interpretation.

## Conclusion

Vasculitides are a group of several diseases affecting the cardiovascular system. These disorders lead to premature atherosclerosis, increasing morbidity and mortality and worsening the prognosis; furthermore, they may involve the heart too, due to the extension of the inflammatory process they cause also in the coronary vessel wall and eventually in the heart, including pericardium, myocardium and conduction system. Cardiovascular events are increased in all the major subtypes of systemic vasculitis. Vascular properties impairment, as measured by PWV, is observed in most subtypes of vasculitis, and in particular during active disease, but evidence of accelerated atherosclerosis is reported in Takayasu's arteritis only, and in ANCA-associated vasculitides. The increased CV event rate observed in patients with vasculitis is likely due to the contribution of active vasculitis, persistent endothelial dysfunction—also causing a prothrombotic state, and accelerated atherosclerosis. Early treatment and adequate control of vasculitis is crucial to minimize inflammation and to prevent vascular damage. The findings summarized in this review underline the importance of an effective treatment of conventional CV risk factors, and call for additional investigation of ways to mitigate the risk excess. How much the anti-inflammatory therapies used to treat rheumatic diseases may improve CV outcomes is unknown so far, and it requires further study. Additional, rigorous, clinical trials are required to develop and validate novel biomarkers to stratify CV risk and to support the intensity of therapy required to improve the prognosis of patients with rheumatologic diseases, also through an evidence-based approach. These strategies should help to avoid complications associated with unnecessary invasive evaluation, prevent over-testing, and minimize imaging radiation exposure, as well as save healthcare resources.

## Data Availability Statement

The raw data supporting the conclusions of this article will be made available by the authors, without undue reservation.

## Author Contributions

AL and GM: conceptualization. LR and PM: methodology. AL, CG, and GM: validation. CM and LR: investigation. CG and PM: data curation. AL, CM, and GM: writing—original draft preparation. CS and GS: writing—review and editing. GM: final supervision. AL and GM: revision and final acceptance. All authors have read and agreed the published version of the manuscript.

## Funding

The APC was funded by IRCCS Neurolesi, Messina.

## Conflict of Interest

The authors declare that the research was conducted in the absence of any commercial or financial relationships that could be construed as a potential conflict of interest.

## Publisher's Note

All claims expressed in this article are solely those of the authors and do not necessarily represent those of their affiliated organizations, or those of the publisher, the editors and the reviewers. Any product that may be evaluated in this article, or claim that may be made by its manufacturer, is not guaranteed or endorsed by the publisher.

## Abbreviations

AAV: Anca associated vasculitis
AIx: augmentation index
ANCA: anti-neutrophil cytoplasmic antibodies
AS: Arterial Stiffness
AUC: area under the curve
baPWV: brachial-ankle pulse wave velocity
BD: Behcet's disease
BMI: body mass index
BNP: B-type natriuretic peptide
BSAS: Behcet's Syndrome Activity Scale
CRP: C-reactive protein
CAD: coronary artery disease
CDAI: clinical disease activity index
CSS: Churg-Strauss syndrome
CV: cardiovascular
CVD: cardiovascular disease
CVE: cardiovascular events
DAS28: disease activity score 28-joints
DBP: diastolic blood pressure
DMARD: disease-modifying anti-rheumatic drugs
ECM: extracellular matrix
EF: ejection fraction
EGPA: eosinophilic granulomatosis with polyangiitis
ESR: erythrocyte sedimentation rate
EULAR: European League Against Rheumatism
GPA: Granulomatosis with Polyangiitis
HDL: high-density lipoprotein
HF: heart failure
HR: heart rate
Hcy: homocysteine
IHD: ischemic heart disease
IL: interleukin
IRD: inflammatory rheumatic diseases
LDD: Long Disease Duration
LDL: low-density lipoprotein
MACE: mayor adverse cardiovascular events
MAP: mean arterial blood pressure
MPA: microscopic polyangiitis
MPO: myeloperoxidase
MTX: methotrexate
MRI: magnetic resonance imaging
PDGF: platelet-derived growth factor
PP: pulse pressure
PR3: proteinase 3
PVAS: Pediatric Vasculitis Activity Score
PWV: pulse-wave velocity
PWV-CF: pulse-wave velocity carotid femoral
PWC-CR: pulse-wave velocity carotid radial
SBP: systolic blood pressure
SDD: short disease duration
SLE: systemic lupus erythematosus
TAK: Takayasu's arteritis
TNF: tumor necrosis factor
US: ultra-sonography
VEGF: vascular endothelial growth factor.
