# Supplementary material for: Arterial Stiffness and Adult Onset Vasculitis: A Systematic Review
Source: Front Med (Lausanne). 2022 May 12;9:824630. doi: 10.3389/fmed.2022.824630 (PMC9133451; doi:10.3389/fmed.2022.824630)
Supplement: Supplementary file 1 [file Table_1.DOCX]

| **Disease** | **Study** | **Mean**  **Duration**  **(months)** | **Numbers** | **M/F** | **Mean Age**  **Years** | **CF-PWV p value** | **CR-PWV p value** | **PWV-brachial** | **PWV-Carotid** | **AI Brachial** | **AIx C** | **AIx R** | **C-Beta index** | **F-Beta Index** | **brachial-ankle PWV p value** | **Aortic %** |
| --- | --- | --- | --- | --- | --- | --- | --- | --- | --- | --- | --- | --- | --- | --- | --- | --- |
| **TAKAYASU** |  |  |  |  |  |  |  |  |  |  |  |  |  |  |  |  |
| 1 | Raninen(6) | 16.4 (4–36) | P: 16  C: 16 | 2/14  2/14 | 45.7 (21-66) 46.4 (25-64) |  |  |  |  |  |  |  | 10.7(6.2±19.8) **0.004** 7.7 (4.2±10.1) | 10.7 (5.1-15.2)  8.6 (4.7±14.7) |  |  |
| 2 | NG(5) |  | P: 10  C: 11 | 0/10  0/11 | 41.±12.5 **0.05**  32.4±5.5 | 12.0±4.3(m/s) **0.03**  8.3±1.1 | 10.4±2.8(m/s) 10.9±1.4 |  |  |  | 40.0±13.8 (%)  **0.02** 26.6±8.7 | 33.1±10.3(%) **0.001** 14.9±9.9 |  |  |  |  |
| 3 | Neto(7) |  | P:27  C:27  Active:15 Remitted:12 HTN YES :18 HYN NO: 9 DD≤ 5 yrs:7. DD>5 yrs:20 VaS Yes: 6 VaS Yes: 21 |  | 32.37 ± 8.26  33.89 ± 10.12 | 9.77 ± 3.49 **0.009** 7.83 ± 1.06 9.89 ± 3.97 9.59 ± 2.80 9.29 ± 3.87 10.72 ± 2.47 8.82 ± 1.49 10.03 ± 3.09 12.40 ± 4.42 **0.03** 9.01 ± 2.87 |  |  |  |  |  |  |  |  |  |  |
| 4 | Liu(2) | 4.5(0.9-14.3)  5.0(1.0-10.0) | P: 72  High baPWV 24 Low baPWV 48 | 10/62  1/23  9/39 | 34.5±11.9 36.6±12.0 33.4±11.9 |  |  |  |  |  |  |  |  |  | 1424.1±431.2 cm/s. 1904.0±408.4 cm/s. **<0.001** 1184.1±151.0 cm/s. |  |
| 5 | Wang(4) |  | P: 48  C: 40 | 12/36  11/29 | 45.0±6.7 44.5±5.6 |  |  |  |  |  |  |  |  |  | 17.0±3.8 m/s **<0.002** 13.8±3.0 m/s |  |
| 6 | Yang (8) |  | P:15  C :15 |  |  | 8.37±2.23. 6.46±1.15 |  |  |  |  |  |  |  |  |  |  |
| 7 | Yurdakul(9) |  | P:33  C:20  SLE: 18 | 2/31  3/17  1/17 | 39.06 ± 11.44  36.7±11.9 42.05±10.42 |  |  |  |  |  |  |  |  |  |  | 5.77±3.2  11.97±6.7  13.91±4.77 |
| 8 | He(1) | 5 (6-120)  58(6-144) 26 (6-117) | P: 240  CVE 74  No CVE: 166 | 40/200  16/58  24/142 | 34 (IQR 24- 44)  37 (26-45) 34(24-44) |  |  |  |  |  |  |  |  |  | 13.35 (IQR 11.31–16.43) m/s 15.05 (12.47-21.26) **<0.001** 12.75 (11.12-14.69) |  |
| 9 | Wang(3) |  | P: 67  C: 67  Inactive TAK  Active TAK | 0/67  0/67  0/24  0/43 | 35.68±10.42**<0.05** 39.67±9.29 42.77±10.09 31.72±10.59 |  |  |  |  |  |  |  |  |  | 1495.55±431.72cm/s **<0.001** 1211.37±154.42 cm/s 1381.75±373.33 cm/s 1553.72±451.76 cm/s |  |
| **ANCA**  **Vasculitis** |  |  |  |  |  |  |  |  |  |  |  |  |  |  |  |  |
| 3 | Wilde(15) | 103 ±79 | P:39  C: 44 | 21/18  18/26 | 56 ±11 51 ±13 | 9.8 ±2.8 9.0 ±2.2 |  |  |  |  |  |  |  |  |  |  |
| 1 | Booth(13) | 3.5  6  (years) | P Active: 15  P Remission:16  Controls:32 | 6/9  8/8  13/19 | 55 ± 4 54 ± 4 54 ± 1 |  |  | 9.2 ± 0.7 **<0.05** 8.0 ± 0.5 **0.03** 7.5 ± 0.3 |  |  |  |  |  |  |  | 31 ± 3 **< 0.05**  19 ± 3 <**0.003**  22 ± 2 |
| 2 | Yildiz (10) |  | P:5  C: 5 | 4/1  4/1 | 45 ± 15.1 44 ± 14.7 | 9.87±1.40(m/s) **0.04** 8.54±0.68 |  |  |  |  |  |  |  |  |  |  |
| 4 | Slot(11) | 2.6(0.6-16.3) | P: 40  C: 38  GPA: 25  MPA: 7  C Strauss: 4  Renal limit: 4 | 22/12  17/21 | 56.4 ± 10.5 54.4 ± 9.7 | 9.87±1.40(m/s) 0.32 9.27±2.15 MAP CORRECTED 9.77±2.41 **0.04** 8.71±1.71 | 8.85±1.38 (m/s) 0.44 8.47 ± 1.88 MAPCORRECTED 8.86 ± 1.41 **0.02**  7.95 ± 1.77 |  |  |  |  |  |  |  |  |  |
| 5 | Chanouza(12) | 6.0 [3.2-12.0] | P: 53  C: 30 | 35/18  14/16 | 69.0[62.8–75.3] 70.5 [66.8–74.0] |  |  |  |  |  |  |  |  |  |  |  |
| 6 | Pacholczak (14) | 4.5 (1–9) | P: 44  C:53 | 21/23  22/31 | 59 (46–65)  48 (43–61) |  |  |  |  |  |  |  |  |  |  | 7.14(4–9.09) 7.4 (6.25–10.34) |
| **BEHCET** |  |  |  |  |  |  |  |  |  |  |  |  |  |  |  |  |
| 6 | Kobacay(30) |  | BD: 33  RA:24  SLE: 22  C: 19 | 26/7  4/20  1/21  10/9 | 42.5 ± 11.5 35.8 ± 11.1 32.7 ± 8.0 36.2 ± 15.0 | 9.23 ± 1.64 9.26 ± 2.16 9.29 ± 2.46 7.53 ± 0.80 |  |  |  |  |  |  |  |  |  |  |
| 1 | Kurum(16) | 6.64±4.5 | P: 14  C: 28 | 10/4  21/7 | 32.1±7.4 27.9±6.1 | 8.4±1.4 8.5±1.1 |  |  |  |  |  |  |  |  |  |  |
| 2 | PROTOGEROU(18) |  | P: 74  P CS+: 27  P CS-: 47  C: 24 | 24/50  6/21  18/29  8/16 | 40.1 (12.5) 37.1 (12.8) 41.5 (12.1) 40.2 (10.3) |  |  |  |  |  |  |  |  |  |  | 17.9±14.9 **11.2±16.4 p<0.008 24.3±13.3** 11.9±18.16 |
| 3 | Tunc(31) | 7.1±4.1 | BD:26  C:20 | 12/14  10/10 | 33±10 33±7 |  |  |  |  |  |  |  |  |  |  | 7.23±5.93 **<0.001** 2.69±0.55 |
| 4 | Rhee(21) | 78.1±57.4 (9-228) | P: 41  Arthritis+: 15  Arthritis-: 26  C: 53 |  | 37.6±7.9  37.1±7.2 |  |  |  |  |  |  |  | **3.26±0.45 0.007** *3.49±0.37 0.010 3.13±0.44* **3.04±0.32** |  |  |  |
| 5 | PROTOGEROU(17) |  | Inactive: 36  Active: 11  C:30 | 21/15  8/3  19/11 | 42.3 ± 13.1 38.8 ± 8.9. 41.9 ± 9.9 |  |  |  |  |  | 23.8 ± 19.3 13.4 ± 12.4 18.0 ± 14.1 |  |  |  |  | 14.5 ± 8.9. 18.3 ± 9.7. 4.5 ± 2.2 |
| 7 | Caldas(22) | 8.9 ± 5.6 10.1 ± 6.1 6.6 ± 4.0 | P:23  Systemic: 15  Mucocutaneous:8  C: 23 | 11/12  10/5  1/7  11/12 | 35.0 ± 7.6 37.2 ± 5.5 30.7 ± 9.5 35.4 ± 6.0 | 8.4 ± 1.1 8.7± 1.2 7.8±0.7 7.5 ± 1.4 |  |  |  |  |  |  |  |  |  |  |
| 8 | Balta(29) |  | P:36  C:35 | 15/20  20/16 | 37.66 ±11.79 35.29± 10.60 |  |  | 7.28±1.42 **0.02**  6.64±0.87 |  |  |  |  |  |  |  |  |
| 9 | Yilmaz(25) | 84.08±89.52 61.02±86.91 | P: 96  Inactive: 53  Active 43  C: 54 | 43/53  24/29  19/24  24/30 | 37.98±11.66 41.19±12.35 34.02±9.50. 40.85±13.59 |  |  | 6.22±1.27  6.86±1.80 |  |  | 21.88±7.97  20.36±6.36  22.65±7.94  24 h AI |  |  |  |  |  |
| 10 | Celik(20) | 73.75±88.65 | P: 96  C: 60 | 43/53  26/34 | 37.98 ± 11.67 35.23 ± 12.58 |  |  |  |  | 21.34 ± 10.62 19.71 ± 11.37 |  |  |  |  |  |  |
| 11 | Yildirim(27) | 9.2±9.4 (years) | P: 30  C: 30 | 17/13  18/12 | 39.6±12.3 37.5±12.7 | 6.35±1.05 5.75±0.83 |  |  |  |  |  |  |  |  |  |  |
| 12 | Yolbaş (24) |  | P: 49  RA: 64  C: 40 | 28/21  51/13  29/11 | 37.3±11.2 50.3±15.1 43.5±13.5 |  |  |  |  |  |  |  | 43.5±13.5 5.8±4.6 4.7±1.2 |  |  |  |
| 13 | Ozdemir(26) | 142 ± 47 | P 68  Group1: 28  Group2: 40  C: 40 | 42/26  18/10  24/16  25/15 | 42.1 ± 8.9  41.3. ± 8.2 |  |  |  |  |  | 3.73 ± 0.45 p<001 3.33 ± 0.24 p<001 3.07 ± 0.17 p<001 |  |  |  |  |  |
| 14 | Ozisler(23) | 11.3 ± 6.5(2-22) | P: 33  Systemic:15  Mucocutaneous:18  C: 33 | 14/19 | 40.4 ± 8.8  40.1 ± 8.5 |  |  |  | **8.3 ± 2.0. p<0.001**  *7.6±1.6 p 0.079*  *8.9±2.2*  **6.5 ± 1.6** |  |  |  | **12.9 ± 6.3 p<0.001** *11.0 ± 4.4 14.6 ± 7.3* **8.1 ± 4.4** |  |  |  |
| 15 | Ayar (19) | 11.5 (0–424) 0 (0–11) 120 (12–424) | P: 54  Short Duration:27  Long Duration: 27  C: 34 | 32/20 17/10 15/12 20/14 | 38.0 (21–61) 35.0 (24–55) 42.0 (21–61) 38.5 (24–60) |  |  | 5.8 (4.6–7.7) **5.6 (4.6–7.5) 0.03 6.40 (5.1–7.7)** 5.75 (4.8–8.8) |  |  |  | **30.0 (4–52) 0.005** 29.00 (7–42) 33.00 (4–52) **22.5 (2–38)** |  |  |  |  |
| 16 | Zencirkiran Agus(28) | 3.23±2.31 | P: 50  C:49 | 40/10  31/18 | 35 (18-61) 36 (18-59) | 9.09 (5.63-14.55**)**  8.52 (5.57-11.7)**0.003** |  |  |  |  |  |  |  |  |  |  |
