# Supplementary material for: Arterial Stiffness and Adult Onset Vasculitis: A Systematic Review
Source: Front Med (Lausanne). 2022 May 12;9:824630. doi: 10.3389/fmed.2022.824630 (PMC9133451; doi:10.3389/fmed.2022.824630)
Supplement: Supplementary file 2 [file Data_Sheet_1.PDF]

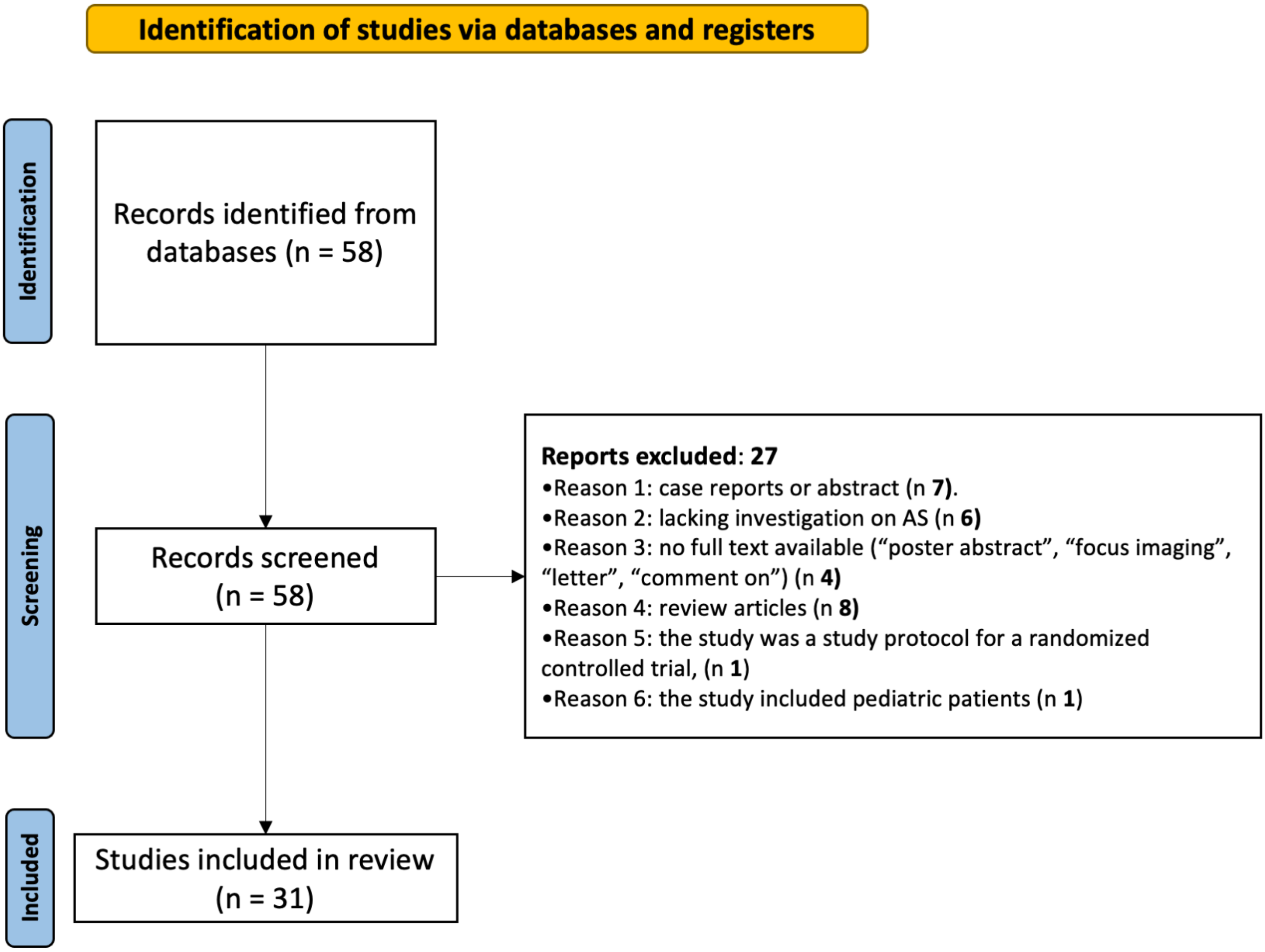

For more information, visit: http://www.prisma-statement.org/
